# Influence of antipsychotics on metabolic syndrome risk in patients with schizophrenia

**DOI:** 10.3389/fpsyt.2022.925757

**Published:** 2022-07-25

**Authors:** Aleksandra Koricanac, Aleksandra Tomic Lucic, Mirjana Veselinovic, Danijela Bazic Sretenovic, Gorica Bucic, Anja Azanjac, Olivera Radmanovic, Mirjana Matovic, Marijana Stanojevic, Aleksandra Jurisic Skevin, Bojana Simovic Markovic, Jelena Pantic, Nebojša Arsenijevic, Gordana D. Radosavljevic, Maja Nikolic, Nenad Zornic, Jelena Nesic, Nemanja Muric, Branimir Radmanovic

**Affiliations:** ^1^Department of Internal Medicine, General Hospital Kraljevo, Kraljevo, Serbia; ^2^Faculty of Medical Sciences, University of Kragujevac, Kragujevac, Serbia; ^3^Department of Internal Medicine, Faculty of Medical Sciences, University of Kragujevac, Kragujevac, Serbia; ^4^Clinic for Rheumatology and Allergology, University Clinical Center Kragujevac, Kragujevac, Serbia; ^5^Department of Biochemistry, Faculty of Medical Sciences, University of Kragujevac, Kragujevac, Serbia; ^6^Department of Laboratory Diagnostics, University Clinical Center Kragujevac, Kragujevac, Serbia; ^7^Department of Physical Medicine and Rehabilitation, Faculty of Medical Sciences, University of Kragujevac, Kragujevac, Serbia; ^8^Center for Molecular Medicine and Stem Cell Research, Faculty of Medical Sciences, University of Kragujevac, Kragujevac, Serbia; ^9^Department of Physiology, Faculty of Medical Sciences, University of Kragujevac, Kragujevac, Serbia; ^10^Department of Surgery, Faculty of Medical Sciences, University of Kragujevac, Kragujevac, Serbia; ^11^Department for Anesthesiology and Reanimation, University Clinical Center Kragujevac, Kragujevac, Serbia; ^12^Clinic for Endocrinology, University Clinical Center Kragujevac, Kragujevac, Serbia; ^13^Department of Psychiatry, Faculty of Medical Sciences, University of Kragujevac, Kragujevac, Serbia; ^14^Psychiatry Clinic, University Clinical Center Kragujevac, Kragujevac, Serbia

**Keywords:** schizophrenia, cytokine, metabolic syndrome, risperidone, clozapine, aripiprazole

## Abstract

**Objective:**

Many studies so far have shown that antipsychotic therapy may have an effect on the development of metabolic syndrome in patients diagnosed with schizophrenia. Our goal was to determine whether our respondents are at risk for developing metabolic syndrome and who is more predisposed to it.

**Methods:**

In a stable phase, 60 patients diagnosed with schizophrenia were equally divided into three groups according to the drug (risperidone, clozapine, and aripiprazole monotherapy). Control group had 20 healthy examinees. Patients were evaluated first using The Positive and Negative Syndrome Scale (PANSS). Prolactin, lipid status, glycemia, insulin, cytokine values (IL-33, TGF-β, and TNF-α) and C-reactive protein (CRP) were measured. Also, Body mass index (BMI), Homeostatic Model Assesment for Insulin Resistance (HOMA index), waist and hip circumference (WHR) and blood pressure (TA) measurement were performed in the study.

**Results:**

Patients treated with risperidone compared to healthy control subjects and aripiprazol group of patients had statistically significant difference in prolactin levels. In clozapine group compared to healthy control group values of HDL cholesterol and glucose level were statistically significant different. In aripiprazole group compared to healthy control group value of BMI was statistically significant different. Statistically significant correlations were found in TNF-α with glucose and HOMA index in risperidone treated patients and with BMI in clozapine group of patients; IL-33 with glucose in risperidone and with BMI in clozapine group of patients and TGF-β with glucose in risperidone group, with insulin and HOMA index in clozapine group and statistically significant negative correlation with LDL cholesterol in aripiprazole group of patients.

**Conclusion:**

Patients on risperidone and clozapine therapy may be at greater risk of developing metabolic syndrome than patients treated with aripiprazole. Statistically significant difference in concentration of TNF-α and TGF-β was in the group of patients treated with risperidone compared to healthy control group.

## Introduction

Schizophrenia is a serious illness in psychiatry. The disease itself is accompanied by numerous changes in the functioning of these people in everyday life. Then, treatment with antipsychotics in patients with schizophrenia is often with side effects. It is also inevitable to monitor the immune response, activation of the inflammatory process, how it maintains and regulates itself in patients. Hence, it is very difficult to approach health improvement conditions of these patients, choose the appropriate antipsychotic and then, as far as possible, influence the adverse effects of the therapy itself and other associated factors that may contribute to it.

When we take into account previous research, we see that patients are at risk of developing diabetes, heart disease and changes in bone density, both due to therapy and its side effects and due to the lifestyle of these patients (1–4). Also, the role of cytokines, immune markers, is increasingly present in research in patients with schizophrenia. Determining the role of individual interleukins in different phases and during the disease itself is of great importance for further approach not only in the diagnosis, but also in the treatment of schizophrenia (5–9).

In this study, we have tried to point out certain differences in the treatment of antipsychotics, specifically those treated with risperidone, clozapine and aripiprazole, both in relation to healthy subjects and in their relationship with each other. That side effects in terms of metabolic disorders are less common in patients treated with aripiprazole. To determine the possible connection of cytokines with the examined variables and the clinical symptomatology according to the used antipsychotic. As well as the presence and relationship of cytokines (IL-33, TGF-β, and TNF-α) in the mentioned groups, taking into account that all respondents were in a stable phase of schizophrenia.

## Materials and methods

### Participants

The research study included patients diagnosed with schizophrenia at the Clinic of Psychiatry, University of Kragujevac, according to The Diagnostic and Statistical Manual of Mental Disorders, Fifth Edition (DSM-5). Structured Clinical Interview for DSM (SCID) was used to confirm the diagnosis of schizophrenia and in the control group to exclude the existence of any mental illness. Patients on risperidone, clozapine and aripiprazole therapy were selected. The total number of respondents was *n* = 60, equally divided by the mentioned groups of drugs. The condition to be in the group of subjects diagnosed with schizophrenia was that they had been on antipsychotic therapy for more than 6 months. The control group of *n* = 20 healthy subjects corresponded to the group of patients by age and gender. Subjects in the control group had not previously suffered from any psychiatric disorder, nor had such a family member. In all participants, the presence of cardiovascular, endocrinological, immunological and systemic diseases was excluded and thus the absence of therapy that may effect on bone metabolism or prolactin levels in the blood (antihypertensive, glucocorticoid, methotrexate and other immunosuppressant, antiepileptic therapy, etc.). Both the subjects diagnosed with schizophrenia and the control group respondents were persons over 18 years of age. Participants were introduced to the course of the study and signed written informed consent to participate after being given the explanation. The study was approved by the Ethics Committee.

### Clinical assessments

All patients were assessed for clinical symptoms of schizophrenia using SCI-PANSS-The Positive and Negative Syndrome Scale (PANSS).

### Laboratory measurements

Biochemical analyses were done for all subjects, laboratory analysis that included determination of: prolactin, cholesterol, triglycerides, HDL cholesterol, LDL cholesterol, glycemia, insulin, C- reactive protein (CRP), all at 8 a.m., as well as serum cytokine concentrations of IL-33, TGF-β, and TNF-α (IL, Interleukin; TNF, Tumor Necrosis Factor; TGF, Transforming growth factor). Cytokine concentrations (IL-33, TGF-β, and TNF-α) were measured by ELISA according to the established manufacturer’s protocol (R&D Systems, Minneapolis, MN, United States).

### Measurements and indices made in the study

Body mass index (BMI), Homeostatic Model Assesment for Insulin Resistance (HOMA index) and waist and hip circumference (WHR) and blood pressure (TA), were performed in the study.

BMI is calculated by dividing the subject’s weight by the height in square meters. Based on the obtained value, we can assess the degree of obesity or malnutrition according to certain categories or consider as normal weight (Underweight is <18.5; Normal weight is in a range 18.5–24.9; Overweight is in a range 25–29.9; Obesity BMI is of 30 or greater).

The HOMA index is used to assess the existence of insulin resistance, based oncalculating the values of insulin and glucose concentration. Higher value of this index indicates the existence of insulin resistance (cut-off value is >2).

The waist-to-hip ratio (WHR) allows us to establish the distribution of fat and thus determine whether we are a certain health risk (WHR of 1.0 or higher increases the risk for all obesity- related diseases).

Blood pressure (TA) is measured with a certain device, the obtained values of systolic and diastolic blood pressure are expressed in millimeters of mercury (mm Hg). By measuring blood pressure, and in accordance with the obtained values, we can determine whether patients are in the risk of developing cardiovascular disease and stroke in the first place (normal TA is when systolic pressure is less than 120 mmHg and diastolic pressure is less than 80 mmHg).

### Statistical analysis

Statistical data processing was performed in IBM SPSS Statistics v.20. Data are presented descriptively using mean and standard deviation or median for continuous variables and using absolute and relative frequencies for categorical variables. The normality of the data distribution was analyzed using the Shapiro-Wilk normality test. For the analysis of continuous variables (age, PANSS, metabolic variables, cytokines, and cytokine ratio) in relation to groups of subjects according to the drug they use, one-factor ANOVA was used for different groups if data followed normal distribution or Kruskal–Wallis test if data did not follow normal distribution. For the analysis of continuous variables with each other, the method of linear correlation was applied, Pearson’s correlation coefficient was interpreted if the data followed the normal distribution or Spearman’s correlation coefficient if the data did not follow the normal distribution. To analyze the gender of the respondents in relation to the groups of respondents, according to the drug they use, the chi-square test for independence was applied.

The results were considered statistically significant if the *p*-value was less than or equal to 0.05. In the case of subsequent tests, since four groups were compared with each other, that we had six subsequent tests, we considered the results statistically significant if the *p*-value was less than 0.0083.

### Strength of study

Using G-Power software (G*Power 3.1.2), and based on a certain sample size of 20 respondents per group, the strength of the study is 82.44%.

## Results

### Demographic characteristics and clinical scale for the assessment and clinical monitoring of the course of schizophrenia and pharmacotherapeutic response, scales of positive and negative symptoms of schizophrenia (Positive and Negative Syndrom Scale)

*n* the group of patients treated with risperidone participated *n* = 15 male and *n* = 5 female respondents (75.0% male/25.0% female respondents). In the group with clozapine in treatment there were *n* = 9 male and *n* = 11 female respondents (45.0% male/55.0% female respondents). In the third group of patients treated with aripiprazole there were *n* = 6 male and female *n* = 14 respondents (30.0% male/70% female respondents). When it comes to the control group, it consisted of *n* = 8 male and *n* = 12 female subjects (35.0% male/65% female subjects). We calculated the magnitude of the impact using Cramer’s V coefficient, which was 0.350 and indicated a strong impact. It was observed that only in the group using Risperidone were more men while in the group using Aripiprazole and in the control group more women.

In the group of patients treated with risperidone mean age (SD) was 43.70 ± 7.61, in clozapine group mean age (SD) was 45.65 ± 12.24, in aripiprazol treated patients mean age (SD) was 40.60 ± 12.77 and in health control group mean age (SD) was 44.50 ± 9.83. There was no statisticaly significant difference between any mentioned groups in mean age (Table 1).

**TABLE 1:** Demographic data of the participants enrolled in the study.

Gender	Risperidone (Percent)	Clozapine (Percent)	Aripiprazole (Percent)	Health control group (Percent)	Chi-Square						
Male	75.0	45.0	30.0	35.0	**0.020 (Cramer’s *V* = 0.350)**						
Female	25.0	55.0	70.0	65.0							
**Age**	**Risperidone (Mean ± SD)**	**Clozapine (Mean ± SD)**	**Aripiprazole (Mean ± SD)**	**Health control group (Mean ± SD)**	**ANOVA**	**p^**a**^**	**p^**b**^**	**p^**c**^**	**p^**d**^**	**p^**e**^**	**p^**f**^**
Age	43.70 ± 7.61	45.65 ± 12.24	40.60 ± 12.77	44.50 ± 9.83	0.489	0.941	0.801	0.995	0.456	0.987	0.665

The Shapiro-Wilk normality test was used to check the normality of the data distribution. For all four groups of data, we conclude that the data follow the normal distribution. Since the data follow a normal distribution, for age in relation to the defined groups of subjects we use a parametric test, a one-factor ANOVA for different groups with subsequent tests. Using the chi-square test for independence, we found that the gender of the respondents and the group to which they belong (the drug they use) are dependent characteristics (*p* < 0.05). To assess the “strength” of this result, we calculated the magnitude of the impact of using Cramer’s V coefficient, which is 0.350 and indicates a strong impact.

p^*a*^: Risperidone vs. Clozapine group; p^*b*^: Risperidone vs. Aripiprazole group; p^*c*^: Risperidone vs. health control group; p^*d*^: Clozapine vs. Aripiprazole group; p^*e*^: Clozapine vs. health control group; p^*f*^: Aripiprazole vs. health control group.

A clinical scale for the assessment and clinical monitoring of the course of schizophrenia and pharmacotherapeutic response, Scales of positive and negative symptoms of schizophrenia (Positive and Negative Syndrome Scale – PANSS), was used in all subjects with schizophrenia, and compared to one another gave us the results that are shown in next table (Table 2). As we can see in Table 2, results in each group of used antipsychotics and PANSS scale were not statistically significant.

**TABLE 2:** Comparison of PANSS between the mentioned groups of drugs.

	Risperidone (Median or Mean ± SD)	Clozapine (Median or Mean ± SD)	Aripiprazole (Median or Mean ± SD)	ANOVA or Kruslal-Wallis	p^a^	p^b^	p^c^
PANSS Total	68.5	67.0	68.0	0.630	0.363	0.516	0.714
PANSS Positive	16.5	15.0	15.0	0.333	0.174	0.233	0.933
PANSS Negative	15.0	14.0	15.0	0.353	0.166	0.304	0.705
PANSS General	39.10 ± 7.43	37.50 ± 5.87	38.55 ± 7.32	0.759	0.745	0.966	0.881

The Shapiro-Wilk normality test. One-factor ANOVA or Kruskal-Wallis test was used. For subsequent tests within ANOVA, post-processing with the Tukey test was applied. Mann-Whitney *U* test. Bonferroni correction (we will consider the results of subsequent tests to be statistically significant if the *p* value is less than 0.017). In the part of descriptive statistical analysis, average values and standard deviation for data that follow the normal distribution and median values for data that do not follow the normal distribution are shown.

PANSS, Positive and Negative Syndrome Scale; p^*a*^: Risperidone vs. Clozapine group; p^*b*^: Risperidone vs. Aripiprazole group; p^*c*^: Clozapine vs. Aripiprazole group.

However, the results we obtained in the correlation between the cytokines and PANSS scale according to each group of used antipsychotics are as follows (Table 3).

**TABLE 3:** Correlation of PANSS and measured cytokines in each treated group.

	TNF-α			IL-33			TGF-β		
	Risperidone	Clozapine	Aripiprazole	Risperidone	Clozapine	Aripiprazole	Risperidone	Clozapine	Aripiprazole
PANSS Total	Rho = −0.063 P = 0.793	Rho = −0.090 P = 0.707	Rho = 0.603 P = 0.005**	Rho = 0.459 P = 0.042*	R = 0.148 P = 0.533	R = −0.292 P = 0.212	Rho = −0.187 P = 0.430	R = 0.243 P = 0.301	R = −0.255 P = 0.277
PANSS Positive	Rho = 0.145 P = 0.543	Rho = 0.051 P = 0.830	Rho = 0.593 P = 0.006**	Rho = 0.374 P = 0.104	Rho = 0.160 P = 0.500	Rho = −0.306 P = 0.189	Rho = −0.107 P = 0.655	Rho = 0.302 P = 0.196	Rho = −0.223 P = 0.344
PANSS Negative	Rho = −0.055 P = 0.818	Rho = 0.023 P = 0.923	Rho = 0.555 P = 0.011*	Rho = 0.431 P = 0.058*	Rho = 0.258 P = 0.271	R = −0.283 P = 0.227	Rho = −0.231 P = 0.327	Rho = 0.486 P = 0.030*	R = −0.188 P = 0.428
PANSS General	Rho = −0.083 P = 0.729	Rho = 0.019 P = 0.937	Rho = 0.608 P = 0.004**	Rho = 0.344 P = 0.138	R = 0.171 P = 0.471	R = −0.208 P = 0.379	R = −0.233 P = 0.257	R = 0.185 P = 0.435	R = −0.215 P = 0.362

The Shapiro-Wilk normality test. Pearson’s correlation or the Spearman’s correlation coefficient. Pearson’s correlation coefficient is denoted by “R” and Spearman’s correlation coefficient is denoted by “Rho”. If R or Rho is >0.5, it means that there is a positive and strong correlation, and the condition for it to be statistically significant is that p is <0.05.

PANSS, Positive and Negative Syndrome Scale; IL, Interleukin; TNF, Tumor Necrosis Factor; TGF, Transforming growth factor.

In the group of patients treated with risperidone, there is a statistically significant correlation of IL-33 with PANSS total score (*p* = 0.042) and PANSS negative (*p* = 0.058) subscale. No other statistically significant correlation was found in other cytokines and PANSS scale in risperidone group of patients.

In the group of patients treated with clozapine, there is a statistically significant correlation between TGF-β and PANSS negative subscale (*p* = 0.030). No other statistically significant correlation was found in other cytokines and PANSS scale in clozapine group of patients.

When it comes to aripiprazole treated patients there is a statistically significant correlation of TNF-α with PANSS total score (*p* = 0.005), PANSS positive (*p* = 0.006), PANSS negative (*p* = 0.011) and PANSS general (*p* = 0.004) subscales. No statistically significant correlation between other cytokines and PANSS scale was found in aripiprazole group of patients.

### Comparison of observed metabolic variables (prolactin level, lipid status measurements, glucose, insulin, body mass index, homeostatic model assesment for insulin resistance index and waist and hip circumference) between examined groups

Comparing all groups we have at our disposal and measured values that can be taken as predictors of metabolic syndrome, we came to the following results shown in Table 4.

**TABLE 4:** Comparison of observed metabolic variables between the groups of patients treated with risperidone, clozapine, and aripiprazole toward the health control group and within them.

	Risperidone (Median or Mean ± SD)	Clozapine (Median or Mean ± SD)	Aripiprazole (Median or Mean ± SD)	Health control group (Median or Mean ± SD)	ANOVA or Kruslal-Wallis	p^a^	p^b^	p^c^	p^d^	p^e^	p^f^
Prolactin(mIU/L)	**500.5**	338.0	**221.5**	**235.0**	0.002**	0.050	0.002**	0.001***	0.050	0.181	0.579
CHOL(mmol/l)	5.90 ± 1.16	5.68 ± 1.36	5.49 ± 1.37	5.90 ± 1.03	0.686	0.947	0.730	1.000	0.962	0.947	0.730
TG(mmol/l)	1.53	1.80	1.26	1.24	0.117	0.449	0.337	0.223	0.130	0.012	0.655
HDL(mmol/l)	1.32	**1.14**	1.38	**1.60**	0.012*	0.064	0.989	0.030	0.239	0.006**	0.021
LDL(mmol/l)	3.64 ± 1.17	3.54 ± 1.14	3.28 ± 0.96	3.60 ± 1.02	0.705	0.990	0.704	0.999	0.865	0.998	0.776
Glucose(mmol/l)	5.10	5.20	5.00	4.75	0.051	0.447	0.472	0.119	0.086	0.006**	0.212
Insulin(mIU/L)	8.85	12.29	15.08	16.70	0.094	0.914	0.051	0.105	0.072	0.083	0.978
BMI(kg/m2)	26.97 ± 4.54	27.03 ± 3.54	**28.85** ± **6.20**	**24.84** ± **3.43**	0.060	1.000	0.566	0.456	0.594	0.431	0.034*
HOMA(mmol/l* mIU/L/22.5)	1.96	2.71	3.41	3.40	0.136	0.507	0.045	0.107	0.110	0.250	0.776
WHR(cm)	0.84 ± 0.03	0.85 ± 0.03	0.85 ± 0.03	0.86 ± 0.03	0.126	0.247	0.337	0.059	0.404	0.236	0.130

The Shapiro-Wilk normality test. One-factor ANOVA or Kruskal-Wallis test was used. For subsequent tests within ANOVA, post-processing with the Tukey test was applied. Mann-Whitney *U* test. Bonferroni correction (we will consider the results of subsequent tests to be statistically significant if the *p* value is less than 0.0083). In the part of descriptive statistical analysis, average values and standard deviation for data that follow the normal distribution and median values for data that do not follow the normal distribution are shown.

CHOL, Cholesterol; TG, Triglycerides; HDL, High density lipoprotein; LDL, Low density lipoprotein; BMI, Body Mass Index; HOMA index, Homeostatic Model Assessment for Insulin Resistance; WHR, Waist-to-hip ratio; p^*a*^: Risperidone vs. Clozapine group; p^*b*^: Risperidone vs. Aripiprazole group; p^*c*^: Risperidone vs. health control group; p^*d*^: Clozapine vs. Aripiprazole group; p^*e*^: Clozapine vs. health control group; p^*f*^: Aripiprazole vs. health control group.

The median value of prolactin level was 500.5 in the group of patients treated with risperidone and 235.0 in the control group, thus this difference was statistically significant (*p* = 0.001).

The median value of HDL cholesterol was 1.14 in the group of patients treated with clozapine, and 1.60 in the control group, so this difference was statistically significant (*p* = 0.006). The median glucose level was 5.20 in the group of patients treated with clozapine and 4.75 in the control group, and this difference was statistically significant (*p* = 0.006).

Mean ± SD value of BMI was 28.85 ± 6.20 in the aripiprazole group of patients and 24.84 ± 3.43 in the control group, thus this difference was statistically significant (*p* = 0.034).

No other statistically significance differences were found in the values of all others variables between the patients in each drug group and subjects in the control group.

Comparison within groups of patients on the mentioned therapy in observed metabolic variables showed the difference only in measured levels of prolactin.

In risperidone group median value of prolactin level was 500.5 and in aripiprazole group of patients 221.5, so the difference was statistically significant (*p* = 0.002).

### Correlation of observed metabolic variables and cytokines

When it comes to the measured values of cytokines in these three groups based on used therapy and according to the already mentioned observed variables in the study, we came to the following results in correlation within them (Table 5).

**TABLE 5:** Correlation of observed metabolic variables and measured cytokines in each group of patients.

	TNF-α			IL-33			TGF-β		
	Risperidone	Clozapine	Aripiprazole	Risperidone	Clozapine	Aripiprazole	Risperidone	Clozapine	Aripiprazole
Prolactin (mIU/L)	Rho = 0.178 P = 0.452	Rho = −0.061 P = 0.799	Rho = −0.093 P = 0.698	Rho = −0.190 P = 0.423	Rho = 0.100 P = 0.675	Rho = 0.149 P = 0.531	Rho = 0.006 P = 0.979	Rho = 0.373 P = 0.105	Rho = 0.211 P = 0.371
Cholesterol (mmol/l)	Rho = −0.002 P = 0.995	Rho = −0.096 P = 0.686	Rho = −0.063 P = 0.791	Rho = 0.034 P = 0.920	R = −0.019 P = 0.935	R = −0.239 P = 0.311	Rho = 0.050 P = 0.835	R = 0.316 P = 0.175	R = −0.351 P = 0.129
Triglycerides (mmol/l)	Rho = 0.208 P = 0.378	Rho = 0.169 P = 0.477	Rho = 0.173 P = 0.466	Rho = 0.240 P = 0.308	R = −0.061 P = 0.799	Rho = 0.033 P = 0.890	Rho = 0.389 P = 0.090	R = 0.376 P = 0.102	Rho = −0.075 P = 0.753
HDL(mmol/l)	Rho = −0.212 P = 0.369	Rho = −0.208 P = 0.379	Rho = −0.114 P = 0.634	Rho = −0.190 P = 0.421	Rho = −0.050 P = 0.833	R = 0.190 P = 0.424	Rhov−0.427 Pv0.061	Rho = −0.029 P = 0.905	R = 0.323 P = 0.165
LDL(mmol/l)	Rho = −0.063 P = 0.791	Rho = −0.086 P = 0.719	Rho = −0.128 P = 0.591	Rho = 0.025 P = 0.917	R = −0.170 P = 0.474	R = −0.383 P = 0.096	R = 0.057 P = 0.810	R = 0.227 P = 0.336	**R = −0.525** P = 0.018*
Glucose(mmol/l)	**Rho = 0.515** P = 0.020*	Rho = 0.161 P = 0.497	Rho = 0.026 P = 0.914	**Rho = 0.700** P = 0.001***	Rho = 0.439 P = 0.053	Rho = −0.084 P = 0.724	**Rho = 0.431** P = 0.028*	Rho = 0.227 P = 0.336	Rho = −0.333 P = 0.152
Insulin(mIU/L)	Rho = 0.223 P = 0.345	Rho = 0.128 P = 0.591	Rho = 0.336 P = 0.150	Rho = −0.038 P = 0.875	R = 0.001 P = 0.998	Rho = −0.075 P = 0.753	Rho = 0.154 P = 0.516	**R = 0.615** P = 0.004**	Rho = −0.095 P = 0.691
BMI(kg/m2)	Rho = 0.069 P = 0.772	**Rho = 0.685** P = 0.001***	Rho = −0.150 P = 0.527	Rho = −0.001 P = 0.997	**R = 0.623** P = 0.003**	R = −0.129 P = 0.588	R = 0.031 P = 0.897	R = 0.307 P = 0.188	R = −0.302 P = 0.196
HOMA(mmol/l* mIU/L/22.5)	**Rho = 0.499** P = 0.025*	Rho = 0.229 P = 0.330	Rho = 0.302 P = 0.196	Rho = 0.120 P = 0.615	Rho = 0.012 P = 0.960	Rho = −0.108 P = 0.649	Rho = 0.310 P = 0.184	**Rho = 0.654** P = 0.002**	Rho = −0.157 P = 0.508
WHR (cm)	Rho = −0.171 P = 0.470	Rho = 0.120 P = 0.615	Rho = −0.140 P = 0.556	Rho = 0.333 P = 0.152	Rho = 0.172 P = 0.581	Rho = −0.247 P = 0.294	Rho = −0.347 P = 0.134	Rho = 0.105 P = 0.565	Rho = −0.151 P = 0.526

The Shapiro-Wilk normality test. Pearson’s correlation coefficient or Spearman’s correlation coefficient. Pearson’s correlation coefficient is denoted by “R” and Spearman’s correlation coefficient is denoted by “Rho”. If R or Rho is > 0.5, it means that there is a positive and strong correlation, and the condition for it to be statistically significant is that p is <0.05.

HDL, High density lipoprotein; LDL, Low density lipoprotein; BMI, Body Mass Index; HOMA index, Homeostatic Model Assessment for Insulin Resistance; WHR, Waist-to-hip ratio; IL, Interleukin; TNF, Tumor Necrosis Factor; TGF, Transforming growth factor.

In the risperidone group of patients, there was a statistically significant correlation in TNF-α with glucose (*p* = 0.020) and HOMA index (*p* = 0.025), between IL-33 and glucose (*p* = 0.001) and TGF- β with glucose (*p* = 0.028).

In the clozapine group of patients, there was a statistically significant correlation between TNF-α and BMI (*p* = 0.001), IL-33 and BMI (*p* = 0.003) and TGF-β with insulin (*p* = 0.004) and HOMA index (*p* = 0.002).

In the aripiprazole group, there was one statistically significant negative correlation between TGF-β and LDL cholesterol (*p* = 0.018).

No other significant correlation was found with any other metabolic variables measured and cytokines in these three groups.

### Comparison of cytokine concentration values of IL-3, TGF-β, and TNF-α

Our study was done in the stable phase of schizophrenia. Results of the cytokine concentration values measured in this study are compared within each of examined groups (Table 6).

**TABLE 6:** Comparison of cytokine concentration values according to all examined groups.

	Risperidone (Median or Mean ± SD)	Clozapine (Median or Mean ± SD)	Aripiprazole (Median or Mean ± SD)	Health control group (Median)	ANOVA or Kruslal-Wallis	p^a^	p^b^	p^c^	p^d^	p^e^	p^f^
TNF-α (pg/ml)	**7.14**	7.20	7.22	**4.50**	0.024	0.695	0.935	0.008**	0.561	0.037	0.009
IL-33 (pg/ml)	79.14	84.83	72.67	82.50	0.660	0.914	0.310	0.507	0.256	0.871	0.499
TGF-β (pg/ml)	**210.5** ± **113.5**	275.1 ± 106.1	266.9 ± 117.7	**303.6** ± **102.4**	0.032	0.237	0.334	0.018*	0.997	0.680	0.552

The Shapiro-Wilk normality test. One-factor ANOVA or Kruskal-Wallis test was used. For subsequent tests within ANOVA, post-processing with the Tukey test was applied. Mann-Whitney *U* test. Bonferroni correction (we will consider the results of subsequent tests to be statistically significant if the *p* value is less than 0.0083). In the part of descriptive statistical analysis, average values and standard deviation for data that follow the normal distribution and median values for data that do not follow the normal distribution are shown.

IL, Interleukin; TNF, Tumor Necrosis Factor; TGF, Transforming growth factor; p^*a*^: Risperidone vs. Clozapine group; p^*b*^: Risperidone vs. Aripiprazole group; p^*c*^: Risperidone vs. health control group; p^*d*^: Clozapine vs. Aripiprazole group; p^*e*^: Clozapine vs. health control group; p^*f*^: Aripiprazole vs. health control group.

In risperidone group of patients compared to the subjects in control group there was a statistically significant difference found in the values of the concentration in two cytokines, TNF-α (*p* = 0.008) and TGF-β (*p* = 0.018).

There are no other statistically significant differences found in any other comparisons according to the given groups and cytokines.

### Cytokine ratio of TGF-β/IL-33, TGF-β/TNF-α, and IL-33/TNF-α in all mentioned groups

We also determined the ratio of these cytokines in all examined groups. The ratio of anti- inflammatory cytokines to pro-inflammatory cytokines (TGF-β/IL-33 and TGF-β/TNF-α) and the interrelationship of proinflammatory cytokines (IL-33/TNF-α) gave the following results (Table 7).

**TABLE 7:** Comparison of cytokines ratio according to all examined groups.

	Risperidone (Median)	Clozapine (Median)	Aripiprazole (Median)	Health control group (Median)	ANOVA or Kruslal-Wallis	p^a^	p^b^	p^c^	p^d^	p^e^	p^f^
TGF-β/ IL-33 (pg/ml)	**0.95**	3.39	3.59	**4.16**	0.003	0.040	0.011	0.001***	0.365	0.062	0.160
TGF-β/ TNF-α (pg/ml)	**21.23**	34.20	23.01	**66.96**	0.001	0.013	0.302	0.000***	0.457	0.031	0.009
IL-33/ZTNF-α (pg/ml)	9.88	9.34	8.34	18.94	0.110	0.978	0.204	0.110	0.262	0.152	0.032

The Shapiro-Wilk normality test. One-factor ANOVA or Kruskal-Wallis test was used. Mann-Whitney *U* test. Bonferoni correction (we will consider the results of subsequent tests to be statistically significant if the *p* value is less than 0.0083). In the part of descriptive statistical analysis, the median values are shown because the data do not follow the normal distribution.

IL, Interleukin; TNF, Tumor Necrosis Factor; TGF, Transforming growth factor; p^*a*^: Risperidone vs. Clozapine group; p^*b*^: Risperidone vs. Aripiprazole group; p^*c*^: Risperidone vs. health control group; p^*d*^: Clozapine vs. Aripiprazole group; p^*e*^: Clozapine vs. health control group; p^*f*^: Aripiprazole vs. health control group.

Risperidone group of patients compared to healthy control group difference was statistically significant in TGF-β/IL-33 ratio (*p* = 0.011) and in TGF-β/TNF-α ratio (*p* = 0.000).

There are no statistically significant differences in all other comparisons according to the given groups and the ratio of these cytokines.

Values of CRP in each patient of mentioned groups and of course in health control group were within the reference value. Blood pressure values measured in all groups were within normal blood pressure values.

In our research patients were taking antipsychotics in the maintenance doses. There were no significant differences in dose range between groups compared on equivalents based on Chlorpromazine. It is provided in Supplementary Material. This was main reason that there was not statistically significant difference in influence on variables (symptomatology, metabolic parameters, and cytokines), between antipsychotic doses, compared on equivalents based on Chlorpromazine. Also, we compared influence of different doses in the same antipsychotic group and there was not statistically significant difference, too.

## Discussion

The therapeutic approach to the treatment of schizophrenia is long-term and involves the use of antipsychotics, which, although necessary in the treatment in addition to the desired effect on the disease itself, may result in an increased risk of metabolic syndrome and cardiovascular events as side effects (10).

Of all the drugs, antipsychotics most often lead to hyperprolactinaemia (10–12). Lacto trophic cells located in the anterior pituitary gland are responsible for the secretion of the hormone prolactin and its synthesis and release are controlled by peptides, neurotransmitters and steroids. Dopamine plays a main inhibitory role on the membrane of lactotroph cells, binding to dopamine D2 receptors. By blocking D2 receptors, antipsychotics eliminate the inhibitory effect of dopamine on prolactin secretion and thus can all cause hyperprolactinaemia. The faster the antipsychotic separates from the D2 receptor, the lower the plasma prolactin increase appears to be. Due to all the listed properties of antipsychotics, we refer to them as prolactin-raising (conventional neuroleptics, amisulpride, risperidone) and prolactin-sparing (clozapine, aripiprazole, olanzapine) antipsychotics (13).

The aspect of the patient in which we establish obesity, high blood pressure values, hyperglycemia, lipid status disorder, and also insulin resistance refer us to the possible metabolic syndrome which should be recognized in order to prevent further cardiovascular adverse events (14, 15). It is known that due to the interaction of antipsychotics with serotonergic, histaminergic and dopaminergic neurotransmitter systems, there is a change in appetite and food intake, which becomes increased (16–18). Antipsychotic therapy is long-lasting and it can lead to weight gain (19). In patients treated with clozapine and olanzapine (except in relation to clozapine) antipsychotics, the risk of metabolic syndrome was significantly higher, and in patients treated with aripiprazole (except in relation to amisulpride) significantly lower than with other antipsychotics (20). It is suggested that hyperprolactinaemia has a role in the pathogenesis of obesity and glucose intolerance and thus can lead to changes in metabolic profile and finally metabolic syndrome (21). Therefore, patients with diagnosed schizophrenia are at higher risk of developing hypertension, cardiovascular disease, diabetes, and osteoporosis (22).

In the present study, statistically significant difference in prolactin level was found in risperidone treated patients compared to the control group and compared to aripiprazole treated patients. One study says that in patients with schizophrenia and hyperprolactinemia adding aripiprazole could be an option to lower its concentration (23). In relation to the gender of the patients, the type of antipsychotics and the duration of the disease, the level of prolactin will also depend (24). Also, one study showed that adjunctive aripiprazole indicated better values of hyperprolactinemia and improvement of clinical symptoms of schizophrenia (25).

It is known that when we say good cholesterol, we think of high-density lipoprotein (HDL). If the values are lower, than considering the anamnesis, clinical history and additional examinations of the patients can help us discover the risk of developing the metabolic syndrome (26). In our study, statistically significant differences in values of HDL cholesterol and glucose level were in clozapine treated patients compared to the control group.

In aripiprazole group of patients compared to healthy control group we found statistically significant difference in BMI value.

Monitoring metabolic parameters in one study in patients on clozapine treatment and long-acting injectable antipsychotics suggested that the first group of patients was at higher risk of developing metabolic syndrome (27). Antipsychotic therapy affects lipid and glycoregulatory disorders, as well as mood swings in patients with schizophrenia (28). Analyzing metabolic disorders as a consequence of the use of antipsychotics, one study indicated that patients treated with olanzapine and clozapine had the worst profile, in contrast to patients treated with aripiprazole, brexpiprazole, cariprazine, lurasidone and ziprasidone (29). The level of HDL cholesterol in overweight patients with schizophrenia differed depending from the antipsychotic they were treated (30).

In patients treated with risperidon a statistically significant positive correlation in TNF-α with glucose levels and HOMA index, IL-33, and TGF-β also with glucose levels was found.

In clozapine group of patients statistically significant correlation of TNF-α and IL-33 with BMI was established and TGF-β with insulin level and HOMA index. It has previously been shown that there is a positive association between IL-1β, IL-6, and TNF-α and prolactin levels in patients with schizophrenia (31). Other study has shown clozapine effect on weight gain and that there was a positive correlation between TNF α and BMI (32). Another study showed expression of certain cytokines (IL-2, IL-6, IL-17, and TNF-α) after chronic clozapine therapy (33).

In aripiprazol treated patients statistically significant negative correlation was between TGF and LDL cholesterol. Without other correlations found in the group of patients treated with aripiprazole, it indicates a lower risk of developing metabolic disorders in this group of patients. It is in accordance with another study which indicates that the use of aripiprazole in patients with milder clinical symptoms or clozapine therapy in refractory patients who have already used antipsychotics should be considered (34).

In obese individuals, we know that occurs a change in the balance between pro-inflammatory (T helper 1 and T helper 17 lymphocytes) and anti-inflammatory (T helper 2 and regulatory T lymphocytes) CD4 + cells. With obesity, and especially in those patients who already have the metabolic syndrome beside, the number of anti-inflammatory regulatory T lymphocytes in adipose tissue decreases (35–37). Based on immunological changes, a recent study indicated that long-term inflammation may be an independent factor in the development of diabetes mellitus type 2 (38). Adiponectin, leptin, resistin, MCP-1, TNF-α, IL-6, IL-1β, IL-10, and TGF-β are the ones that have been most researched so far (39, 40).

Earlier studies have shown that the course of the disease and the effectiveness of therapy are associated with certain immune markers. Thus, an association with acute symptoms in affective disorders and schizophrenia and elevated values of IL-33 has been shown (41). Also, significant association between cognition in patients with schizophrenia and IL-33 levels and the soluble form of IL 33 receptor (sST2) was found (42). In the next study, which examined the values of Gal-3, IL-33, and sST2 in schizophrenia patients, but in different stages of disease, the obtained result indicated that in the phase of exacerbation compared to the control population and patients in remission were higher values of IL -33 and sST2 (43).

In our study in all three groups of respondents risperidone, clozapine and aripiprazole patients, a certain correlation was found among the cytokines (IL-33, TGF-β, and TNF-α) and the SCI- PANSS scale. In the group of patients treated with risperidone, IL-33 significantly correlated with PANSS total score and PANSS negative subscale. In clozapine patients group, TGF-β significantly correlated with PANSS negative subscale. In the group of patients treated with aripiprazole, TNF-α significantly correlated with all subsets of PANSS scale.

One study compared the IFN-γ/TGF-β ratio and the IL-17/TGF-β ratio to the PANSS scale. In the first case, a negative correlation was shown between the mentioned cytokines and the total PANSS score, and in the second case between the mentioned cytokines and the subscale of PANSS negative and PANSS general psychopathology (44). In another study, IL-17 and TGF-β values had a positive correlation with the PANSS total scale (45). In the study we mentioned earlier (43) alongside of observing cytokine values in certain stages of the disease it was examined whether there is a correlation of IL-33 with the PANSS scale. They came to the conclusion that it correlates with the PANSS positive and the PANSS general subscale (43). Other studies have shown a negative correlation of IL-10 with the PANSS scale (positive, negative and total subscale) in chronic schizophrenia patients treated with aripiprazole (34).

At different stages of schizophrenia, the challenge for researchers is to determine which cytokines are more or less present, the ratio of pro-inflammatory to anti-inflammatory cytokines. One study showed that at the very beginning of the disease there was an increased level of both pro-inflammatory and anti-inflammatory cytokines, and later only a prolonged pro-inflammatory response. Of course, more research studies are needed on this topic (46, 47). In one study, they compared the levels of different cytokines in the first episode before drug administration and later after in stable phase treatment. The results indicated that the level of type 2 cytokines that was increased at the beginning, after the therapy with antipsychotics, was reduced (48). It is also explained that in the beginning of the disease due to the pronounced pro-inflammatory response and regulation of chronic inflammation, the presence of anti-inflammatory activity is increased, as it will counteract or limit the first mentioned response (48).

In our study statistically significant difference in TGF-β concentration was found between the risperidone treated patients and the control group. We also founded a statistically significant difference in TNF-α concentration between risperidone and the control group.

TGF-β/IL-33 and TGF-β/TNF-α ratio were statistically significant in risperidone treated patients compared to the control group. There was no statistically significant difference found among any group of subjects in IL-33/TNF-α ratio. Other studies suggest similar results (48, 49). A clear change in the ratio of Th1 and Th2 cells was observed in schizophrenia (50). We have already mentioned that the immune response plays a big role in the disease of schizophrenia and of course it is liable to change after the inclusion of antipsychotics. A higher Th1/Th2 ratio was observed in those patients, but it was weakened after the effect of the therapy. It is also noticed that TGF-β plays a role in attenuating Th1 receptor activity (51).

One study provided an explanation and indicated the role of cytokines in schizophrenia, and thus they could influence the prevention, regulation and treatment of schizophrenia as key molecular targets (52). Cytokines that have changed depending on the clinical status of schizophrenia, so for example the TGF-β marker seemed to be state-related marker and it was increased during acute exacerbation, but after antipsychotic treatment normalized. On the other hand, TNF-α was shown as trait marker, since the levels remained elevated in both of these stages (53). Also, the researchers’ interest is more focused both on examining metabolic and inflammatory changes in patients with chronic schizophrenia (54).

It is necessary to supplement research on the influence of all risk factors, influence and connection among antipsychotics and cytokines. Encourage them to be active, have a hygienic dietary regime and a healthier lifestyle (55–58). Limitations of the study were: limited examined group of patients to the University Clinical Center of Kragujevac (we did not take into account other centers nearby, which would for certain give us a larger number of respondents); cytokine concentration were not measured before antipsychotic treatment; potential introduction of Cariprazine and Paliperidone were not observed, because the number of patients on these drugs was small in our environment.

## Conclusion

Patients treated with risperidone and clozapine may be at greater risk than patients treated with aripiprazole for the development of metabolic syndrome and later on cardiovascular events. This is probably due to the influence of cytokines and their relationship.

In the group of patients treated with risperidone, glucose correlates with all three cytokines, TNF-α, IL-33, TGF-β, while HOMA index correlates with TNF-α. When it comes to clozapine group of patients BMI correlates with TNF-α and IL-33, until HOMA index and insulin correlate with TGF-β.

There is also a difference in the concentration of cytokines TNF-α and TGF-β and cytokine ratio of TGF-β/IL-33 and TGF-β/TNF-α, between patients treated with risperidone and a healthy control group.

Statistically significant difference in prolactin levels between risperidone and aripiprazole treated patients is found.

## Data availability statement

The original contributions presented in the study are included in the article/Supplementary Material, further inquiries can be directed to the corresponding author.

## Ethics statement

The studies involving human participants were reviewed and approved by the Ethics Committee, University Clinical Center Kragujevac. The patients/participants provided their written informed consent to participate in this study.

## Author contributions

All authors were included in the designing of the manuscript, drafting the work, critical revision, and final approval for all aspects of the work, and the final version to be published.
