# Supplementary material for: Influence of antipsychotics on metabolic syndrome risk in patients with schizophrenia
Source: Front Psychiatry. 2022 Jul 25;13:925757. doi: 10.3389/fpsyt.2022.925757 (PMC9357900; doi:10.3389/fpsyt.2022.925757)
Supplement: Supplementary file 1 [file Data_Sheet_1.doc]

| **Medication** | **Equivalents (based on Chlorpromazine)** | **Doses used in study (equivalents based on Chlorpromazine)** |
| --- | --- | --- |
| Chlorpromazine | 100mg |  |
| Risperidone | 1mg | 3-5mg (300-500mg) |
| Aripiprazol | 7.5mg | 15-20mg (200-266mg) |
| Clozapine | 100mg | 200-450mg (200-450mg) |

In our research patients were taking antipsychotics in the maintenance doses. This table shows us that there were no significant differences in dose range between groups compared on equivalents based on Chlorpromazine. This was main reason that there was not statistically significant difference in influence on variables (symptomatology, metabolic parameters and cytokines), between antipsychotic doses, compared on equivalents based on Chlorpromazine. Also, we compared influence of different doses in the same antipsychotic group and there was not statistically significant difference, too.

References

1. Leucht S, Samara M, Heres S, Patel MX, Woods SW, Davis JM. Dose equivalents for second-generation antipsychotics: the minimum effective dose method. Schizophrenia Bulletin. 2014;40(2):314-26. DOI: [10.1093/schbul/sbu001](http://dx.doi.org/10.1093/schbul/sbu001). PubMed PMID: [24493852](http://www.ncbi.nlm.nih.gov/pubmed/24493852); PubMed Central PMCID: [PMC3932104](http://www.ncbi.nlm.nih.gov/pmc/articles/PMC3932104).
2. Leucht S, Samara M, Heres S, et al. Dose equivalents for second-generation antipsychotic drugs: the classical mean dose method. *Schizophr Bull*. 2015;41(6):1397-402.  [[PubMed]](https://www.ncbi.nlm.nih.gov/pmc/articles/PMC4601707/pdf/sbv037.pdf)
3. Patel MX, Arista IA, Taylor M, Barnes TRE. How to compare doses of different antipsychotics: a systematic review of methods. Schizophr Res. 2013;149(1-3):141-8. DOI: [10.1016/j.schres.2013.06.030](http://dx.doi.org/10.1016/j.schres.2013.06.030). PubMed PMID: [23845387](http://www.ncbi.nlm.nih.gov/pubmed/23845387).
4. Rothe PH, Heres S, Leucht S. Dose equivalents for second generation long-acting injectable antipsychotics: The minimum effective dose method. Schizophrenia Res. 2018;193:23-28. DOI: [10.1016/j.schres.2017.07.033](http://dx.doi.org/10.1016/j.schres.2017.07.033). PubMed PMID: [28735640](http://www.ncbi.nlm.nih.gov/pubmed/28735640).
5. Taylor D, Paton C, and Kapur S. The Maudsley Prescribing Guidelines in Psychiatry, Wiley-Blackwell; 11th Edition, 2011.
6. Schatzberg AF, Cole JO, and DeBattista C. Manual of Clinical Psychopharmacology, American Psychiatric Publishing; 7th Edition, 2010.
7. Woods SW. Chlorpromazine Equivalent Doses for the Newer Atypical Antipsychotics. J. Clin. Psychiatry. 2003;64(6):663- 667. DOI: [10.4088/JCP.v64n0607](http://dx.doi.org/10.4088/JCP.v64n0607). PubMed PMID: [12823080](http://www.ncbi.nlm.nih.gov/pubmed/12823080).
